# Two differentially structured collagen scaffolds for potential urinary bladder augmentation: proof of concept study in a Göttingen minipig model

**DOI:** 10.1186/s12967-016-1112-5

**Published:** 2017-01-04

**Authors:** Dorothea Leonhäuser, Katja Stollenwerk, Volker Seifarth, Isabella M. Zraik, Michael Vogt, Pramod K. Srinivasan, Rene H. Tolba, Joachim O. Grosse

**Affiliations:** 1 Department of Urology, RWTH Aachen University Hospital, Pauwelsstraße 30, 52074 Aachen, Germany; 2 FB 9 Department of Biomedical Engineering, Laboratory of Medical and Molecular Biology, Aachen University of Applied Sciences, Jülich, Germany; 3 Interdisciplinary Center for Clinical Research IZKF Aachen, RWTH Aachen University Hospital, Aachen, Germany; 4 Institute for Laboratory Animal Science and Experimental Surgery, RWTH Aachen University Hospital, Aachen, Germany

**Keywords:** Tissue engineering, Urinary bladder, Collagen scaffold, Autologous cell seeding, Large animal model

## Abstract

**Background:**

The repair of urinary bladder tissue is a necessity for tissue loss due to cancer, trauma, or congenital abnormalities. Use of intestinal tissue is still the gold standard in the urological clinic, which leads to new problems and dysfunctions like mucus production, stone formation, and finally malignancies. Therefore, the use of artificial, biologically derived materials is a promising step towards the augmentation of this specialised tissue. The aim of this study was to investigate potential bladder wall repair by two collagen scaffold prototypes, OptiMaix 2D and 3D, naïve and seeded with autologous vesical cells, as potential bladder wall substitute material in a large animal model.

**Methods:**

Six Göttingen minipigs underwent cystoplastic surgery for tissue biopsy and cell isolation followed by implantation of unseeded scaffolds. Six weeks after the first operation, scaffolds seeded with the tissue cultured autologous urothelial and detrusor smooth muscle cells were implanted into the bladder together with additional unseeded scaffolds for comparison. Cystography and bladder ultrasound were performed to demonstrate structural integrity and as leakage test of the implantation sites. Eighteen, 22, and 32 weeks after the first operation, two minipigs respectively were sacrificed and the urinary tract was examined via different (immunohistochemical) staining procedures and the usage of two-photon laser scanning microscopy.

**Results:**

Both collagen scaffold prototypes in vivo had good ingrowth capacity into the bladder wall including a quick lining with urothelial cells. The ingrowth of detrusor muscle tissue, along with the degradation of the scaffolds, could also be observed throughout the study period.

**Conclusions:**

We could show that the investigated collagen scaffolds OptiMaix 2D and 3D are a potential material for bladder wall substitution. The material has good biocompatible properties, shows a good cell growth of autologous cells in vitro, and a good integration into the present bladder tissue in vivo.

## Background

Numerous tissue engineering strategies have been developed over recent years in an attempt to generate a urinary bladder wall substitute that would be applicable for patients suffering from malfunctions, cancer or trauma [1–7]. The experimental investigation of a broad range of differing materials for vesical augmentation reflects the ongoing challenge for sufficient medical treatment concerning malfunctions of the urinary bladder. As the bladder is a complex organ, this aim is hard to achieve. The storage and voiding functions are regulated by an intricate network of different tissues, nerve tracts, transport proteins and receptors [8–10]. Considering the relative complexity of this tissue, it is not surprising that the clinical gold-standard, the usage of absorptive intestinal material, is sub-optimal for long-term treatments, and the need for an equivalent bioengineered alternative apparent [11].

Non-degrading materials such as polytetrafluorethylene (Teflon^®^), silicone or polyurethane were only briefly considered as they led to chronic inflammatory reactions, promoted urinary tract infections, calcifications as well as fistula formation [12–15]. For this reason, mainly biodegradable materials, naturally or artificially derived, came into focus within the last years. Processed tissue such as bladder acellular matrix grafts (BAMG) or commercially available small intestinal submucosa (SIS) have provided a range of different outcomes [4, 6, 15, 16]. Furthermore, SIS was found to induce immune reactions as foreign DNA remnants could be detected in the material. Moreover, the problem of batch-to-batch variations and alterations due to different processing techniques arises and can lead to an instable outcome after the implantation [17–19].

Artificially engineered scaffolds can be designed adjusted for desired task and tissue. Furthermore, a modification with growth factors or the use as drug delivery system is viable and can further enhance ingrowth of the biomaterial into the host tissue. Especially vascular endothelial growth factor (VEGF) showed promising results in some recent studies but also the graftage with recognition patterns for cells like arginine–glycine–aspartic acid (RGD) or isoleucine–lysine–valine–alanine–valine (IKVAV) is feasible [20–23]. Furthermore, the raw materials like polyglycolic acid (PGA), polylactic acid (PLA) or polycaprolactone (PCL) are FDA-approved and commercially available in adequate amounts [24–26]. Drawback of these materials however is that they are foreign to the body and are degraded under generation of acids, which can enhance rejection of the implant [15]. Additionally it has to be granted, that the artificial added molecules are not able to promote tumour development [6].

The use of extracellular matrix-derived components, such as collagen, for scaffold production presents advantages over synthetic polymers and has shown great promise in a number of models of tissue engineering for regenerative medicine [27–31]. Collagen is degraded by enzymes and the degradation products do not interact negatively with the surrounding tissue but, because of its phylogenetic affinity, can be transformed by the organism [6]. In the present study, we selected two differently manufactured collagen type I/III scaffold prototypes, manufactured by Matricel GmbH (Herzogenrath, Germany), after an iterative process of several examinations [32–34]. The company´s main product, a sponge-like collagen scaffold (OptiMaix 3D^®^), has already been used in different approaches of tissue engineering for diverse organs, such as esophagus, cartilage, vascular and smooth tissue, with promising outcomes [26–31]. The aim of this study was to compare the regenerative potential of collagen scaffolds with a relatively simple, planar or sheet-like form (i.e. OptiMaix 2D^®^), with the structurally more complex, orientated porous form (OptiMaix 3D) in a large animal model. Furthermore, the influence of prior cell seeding on scaffold-host integration and bladder tissue repair was also investigated. The employed Göttingen minipig race is a small pig, especially bred for experimental studies. It is widely regarded as an excellent model for urological research because the micturition volume and voiding frequency closely resemble that of humans as we could demonstrate recently. [35]. Here, we present our first data about the OptiMaix 2D and 3D, naïve and seeded with autologous urothelial (UCs) and detrusor smooth muscle cells (SMCs).

## Methods

### Study design

Six adult female Göttingen minipigs aged 17.8 ± 1.0 months with an average weight of 44.8 ± 3.9 kg (mean ± SD) were obtained from a specific pathogen-free (SPF) breeding facility (Ellegaard Göttingen Minipigs A/S, Dalmose, Denmark) and clinically examined by a veterinarian after arrival. All animals were acclimatized for at least 14 days prior to the study.

**Fig. 1:**
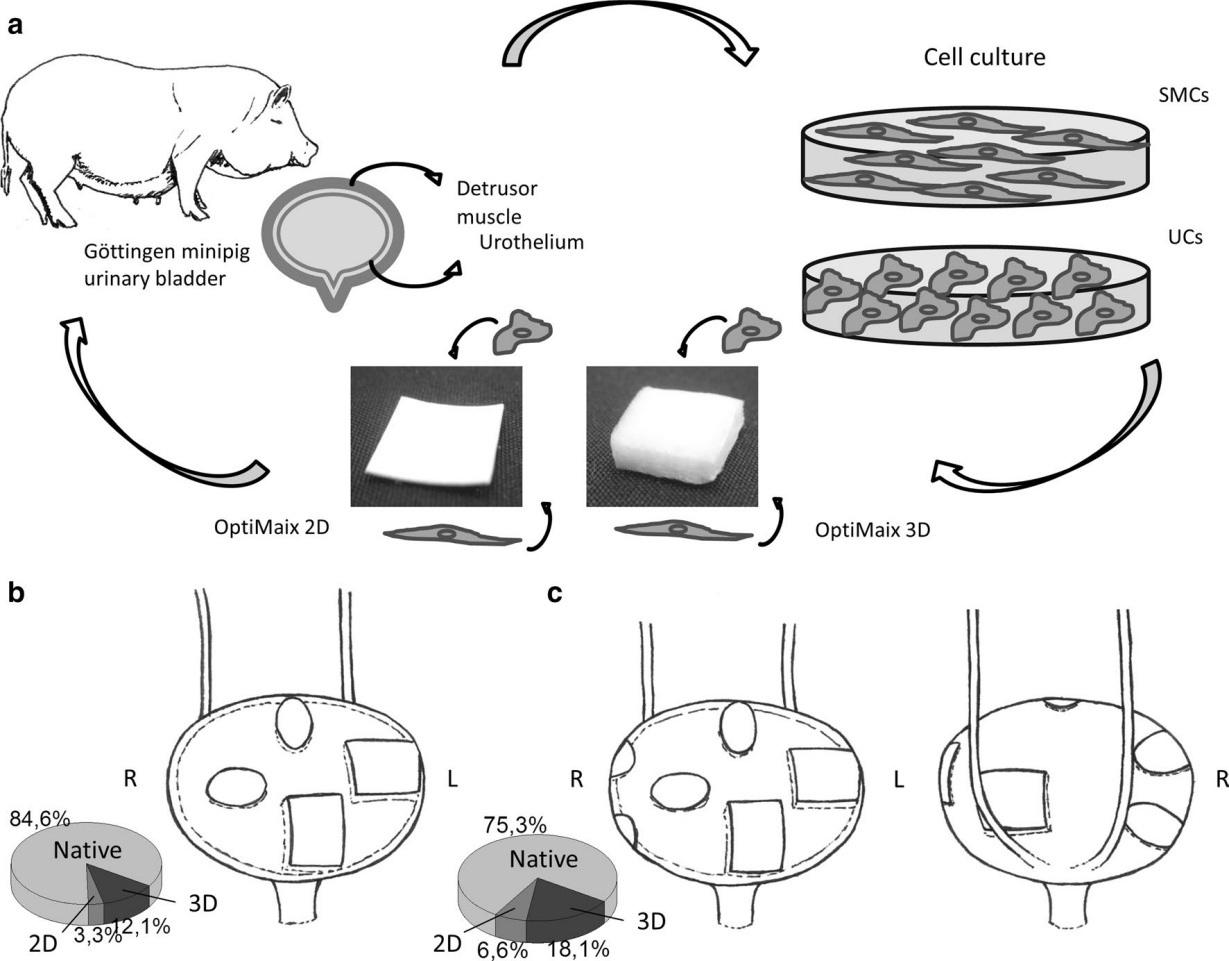
Study design. **a** Isolation and cultivation of primary cells from Göttingen minipig urinary bladder. Seeding of scaffolds was performed after 3 weeks and implantation of seeded scaffolds into the pig bladder after another 3 weeks. **b** During OPI two OptiMaix 2D (*oval*) and 3D (*square*) each, without cells, were implanted into the ventral bladder wall. **c** During OPII, one of the implanted OptiMaix 2D and 3D each, was excised and substituted by a fresh unseeded scaffold of the same make and shape. Two autologously seeded OptiMaix 2D scaffolds were implanted into the right and one autologously seeded OptiMaix 3D scaffold was implanted in the left dorsal bladder wall. After OPI 15.4% and after OPII 24.7% of the native tissue had been substituted by the OptiMaix scaffolds

In a first operation (OPI), two OptiMaix 2D and two OptiMaix 3D without cells were implanted into the ventral urinary bladder wall of a Göttingen minipig. The excised bladder tissue was used for the isolation and tissue culturing of UCs and SMCs (see below). Scaffolds were seeded with autologous tissue cultured UCs and SMCs after 3 weeks, and remained in an incubator for another three weeks prior to the implantation. During operation II (OPII), one of the implanted OptiMaix 2D and 3D scaffolds, was excised and substituted by an appropriate, freshly prepared naïve 2D or 3D scaffold of the same shape. Accordingly, an initial assessment of implant–host integration after 6 weeks was possible. Additionally, two cell-seeded OptiMaix 2D scaffolds were implanted into the right, and one cell-seeded OptiMaix 3D scaffold was implanted into the left dorsal bladder wall (Fig. 1).

**Fig. 2:**
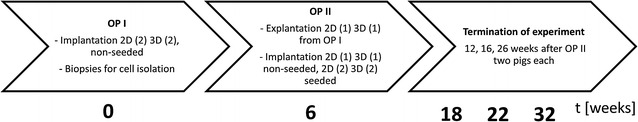
Timeline of the in vivo study. During OPI biopsies were taken for cell cultivation and unseeded scaffolds were implanted. Six weeks later, two scaffolds implanted in OPI were excised for histological evaluation and fresh unseeded as well as autologously seeded scaffolds were implanted. Termination of the experiment was at 18, 22 and 32 after OPI. Number in brackets gives the quantity of implanted scaffolds

At weeks 18, 22 and 32 (i.e. 12, 16 and 26 weeks after OPII), two minipigs were sacrificed and the urinary tract examined. These time points were chosen due to results of two Göttingen minipigs in a preliminary study. The degradation of the scaffolds should have been accomplished at 14–15 weeks after the implantation, according to the assumption of the scaffold’s manufacturer. Instead, pieces of the artificial collagen could still be found. Therefore, one time point before and one after this first time limit, as well as one after a longer period, namely 12, 16 and 26 weeks after OPII were chosen. In addition, by performing two operations in one animal, more information about the timescale of implant-host tissue integration could be obtained in accordance with the 3R-principle. The study design is presented schematically in Fig. 2.

### Surgical procedure

OptiMaix 2D scaffolds were appropriately trimmed to fit the custom made, oval Teflon seeding rings (3.3 cm^2^) immediately before implantation. This pre-cut was performed to have a basis for comparison with the seeded scaffolds, which were implanted in the second operation. The highly porous (pore diameter ≈100 µm) OptiMaix 3D scaffolds proved to be fragile and easily damaged, thus necessitating the use of a larger surface for suturing. The 3D scaffolds were, therefore, implanted and sutured in their delivered format of 3 × 4 cm^2^ (×3.0 mm).

Pre-sedation was performed with an i.m. injection of 4 mg/kg azaperone (Stresnil^®^, Sanochemia Pharmazeutika AG, Neufeld, Austria) and 0.1 mg/kg atropine (Dr. Franz Köhler Chemie GmbH, Bensheim, Germany). A second i.m. injection with 10 mg/kg ketamine (Ceva, Düsseldorf, Germany) was administered after 15 min. Blood samples were taken from all animals for routine blood counts. Anaesthesia was subsequently deepened by an i.v. injection of 1 mg/kg propofol (Fresesnius Kabi, Bad Homburg, Germany) and the animals were intubated. Inhalation anaesthesia was maintained with 1–1.5 vol% of isoflurane (Forene^®^, abbvie, Darmstadt, Germany). A continuous infusion of fentanyl (0.02 mg/kg h, Rotex Medica GmbH, Trittau, Germany) was also administered to ensure analgesia. Additional volume demand during laparotomy was compensated with an infusion of 0.9% sodium chloride solution (B. Braun, Melsungen, Germany) with a rate of 4 ml/kg h.

**Fig. 3:**
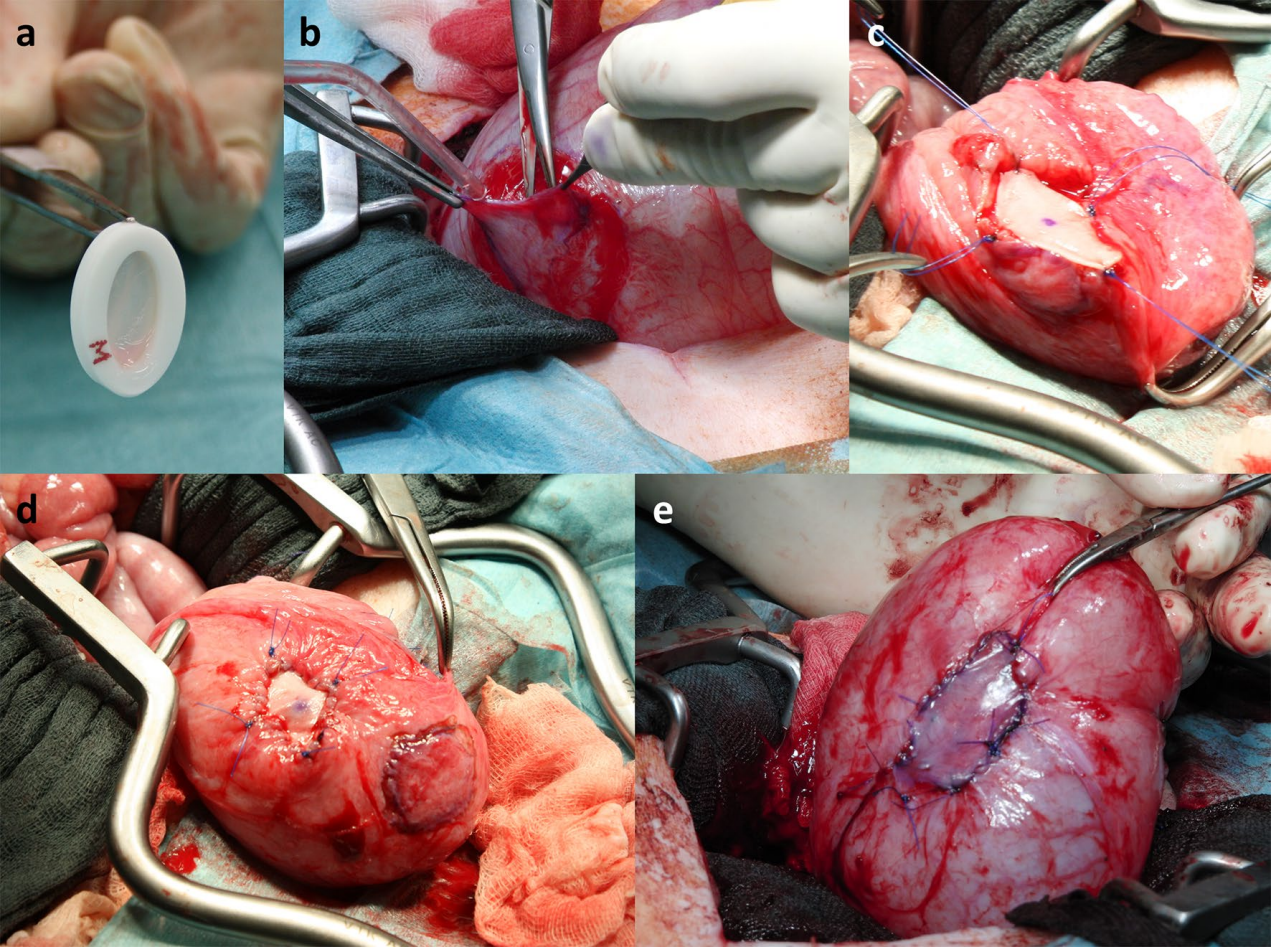
Implantation procedure of a seeded OptiMaix 2D into the minipig bladder. **a** Seeded OptiMaix 2D in the custom made seeding ring. **b** Creation of the serosal flap. **c** Setting of marks with non-degradable sutures. **d** Implanted OptiMaix 2D. **e** Sealed implantation site. Implantation of OptiMaix 3D was performed similarily (not shown)

A midline incision of the lower abdomen was performed after cleaning and disinfection of the operation field. The pigs were provided with a transurethral catheter and the urinary bladder was lifted above the symphysis by filling it with 200 ml 0.9% sodium chloride solution. Urothelial and detrusor muscle tissue of the designated implantation sites was sharp-dissected with scalpel and scissors while simultaneously creating a serosal flap (Fig. 3b). The biopsies, which corresponded in size to that of the intended implants (i.e. 0.8 × 1.4 cm^2^ for the ellipsoid 2D scaffold and 3.0 × 4.0 cm^2^ for the 3D scaffold) were transferred to a sterile cup containing pre-warmed “modified Eagle’s Medium” (MEM, Life Technologies, Braunschweig, Germany) and immediately processed in the cell culture facility (see below). Collagen scaffolds were implanted and secured in the prepared lesion sites with resorbable PDS II 4-0 sutures (Johnson & Johnson, Medical GmbH, Norderstedt, Germany, Fig. 3c). Non-absorbable marking sutures were set with Prolene 3-0 (Johnson and Johnson) at four corners of the scaffold (Fig. 3d) using black suture for the 2D scaffold and blue for the 3D scaffold. The remaining serosal flap was used to cover the scaffold and was fixed with PDS II 4-0 (Fig. 3e). The bladder was then filled with 0.9% sodium chloride solution to check for leakage. The peritoneum, muscle, and subcutis were closed separately with Vicryl 2-0 sutures (Johnson and Johnson) and the skin closed with Prolene 2-0 sutures (Johnson and Johnson). A fresh transurethral catheter was placed to avoid pressure on the healing bladder tissue. Prophylactic antibiotics (Cefuroxime 500 mg, Heumann Pharma GmbH & Co., Nürnberg, Germany) were given twice daily and Carprofen (Rimadyl^®^, 4 mg/kg body weight, Pfizer Deutschland GmbH, Berlin, Germany) was given once daily for analgesia via oral administration for five days.

### Post-operative care

The health status of the animals was checked every day. Assessment of urinary bladder integrity was performed via cystography after one week. For this, minipigs were sedated as described above and up to 350 ml of radiocontrast medium (Conray™, Mallinckrodt pharmaceuticals, Dublin, Ireland) was infused into the bladder via the transurethral catheter. In case of leakage, a fresh transurethral catheter remained in the bladder until a second follow-up after 1 week.

After removal of the catheter, the minipigs were observed in a metabolic cage for 24 h with regular diet and controlled water ad libitum. This monitoring was also done prior to and after each operation as well as the termination of the experiment to evaluate the bladder function. Proof of the time point and volume of the micturition was ensured by video monitoring (Mobotix, Langmeil, Germany) and an urological flow meter consisting of a scale with a collecting vessel and corresponding software (Flow, Laborie, Montreal, Quebec, Canada) as described elsewhere [36].

### Cell culture of UCs and SMCs

Autologous UCs and SMCs were isolated as described previously [37]. Briefly, the urothelium and detrusor muscle were dissected from the bladder biopsy and the cells mechanically dissociated. After gentle agitation (1 h at 37 °C) in MEM with 400 µg/ml collagenase (Liberase^®^, Roche Applied Sciences, Penzberg, Germany) the cell suspension was filtered through a stainless steel mesh (1.0 mm^2^ pore size), washed three times with MEM containing 10% (v/v) fetal calf serum (FCS, Thermo Scientific) and transferred in collagen-coated (Biochrom AG, Berlin, Germany) cell culture flasks (Nunclon™, Thermo Scientific). The UCs were cultured in Keratinocyte-SFM (Life Technologies) and SMCs in a selection medium described elsewhere in a humidified incubator with 5% CO_2_ at 37 °C [32]. The selection medium was used for 3 days to inhibit the growth of fibroblasts in the SMC population. For the subsequent cultivation, SMCs were treated with smooth muscle cell growth medium 2 (Promo Cell GmbH, Heidelberg, Germany). The cultured cells were regularly monitored via a Leica DMI 4000B (Leica Microsystems GmbH, Wetzlar, Germany) and integrated software (Diskus 4.80.5909, Hilgers, Technisches Büro, Königswinter, Germany).

### Cell-seeding and implantation of OptiMaix scaffolds

OptiMaix 2D scaffolds were fixed in the custom made seeding rings and seeded with SMCs. As mentioned above, OptiMaix 3D scaffolds could not as planned be cut to the size of the seeding ring, and therefore, were laid flat in a petri dish and then seeded similarily to the OptiMaix 2D. After 4 h, the petri dishes were filled with MEM containing 20% FCS and 1% amphotericin B and 1% gentamicin. For co-culture, one day later, SMC-seeded scaffolds were placed in a fresh petri dish and UCs were seeded onto the other side of the scaffold. An additional scaffold of each type was seeded to serve as a control for cell growth. Medium was changed every second day.

### Histochemical and immunohistochemical staining of scaffolds

Two times a week (d1 and d4), a piece of 3 mm width of the seeded control scaffolds was cut off and fixed in Carnoy’s Solution [60% ethanol (Merck, Darmstadt, Germany), 30% chloroform, 10% acetic acid (both Carl Roth GmbH & Co.KG, Karlsruhe, Germany)] for 4 h. The fixed samples were dehydrated through ascending concentrations of ethanol, paraffin embedded and cut into 3 µm sections. For subsequent staining, sections were deparaffinized and rehydrated in descending concentrations of ethanol, eventually being washed in PBS. Sections were stained with haematoxylin–eosin (HE), 4′,6-Diamidin-2-phenylindol (DAPI) and nuclear fast red–fast green (RG).

Immunohistochemistry with pancytokeratin (PanCK) and α-smooth muscle actin (α-SMA) was performed as described previously using the antibodies mentioned in Table 1 [37]. Briefly, sections were deparaffinized in descending ethanol solutions. The antigen retrieval was performed using citrate-buffer (Zytomed System GmbH, Berlin, Germany) in a steamer for 30 min, and primary antibodies, diluted in PBS, were incubated for 1 h. The secondary antibody and chromogen development was performed using the DAKO Real EnVision HRP rabbit/mouse with DAB. Images of the stainings were generated using a Leica DM6000B with external light source for fluorescence excitation Leica EL6000 and integrated software (Diskus 4.80.5909, Hilgers, Technisches Büro, Königswinter, Germany).

**Table 1:** Antibodies for immunohistochemical staining of control scaffolds and excised bladder biopsies

Antibody	Clone number	Dilution	Company
α-Smooth muscle actin	1A4	1:400	Dako GmbH, Hamburg, Germany
PanCK	AE1/AE3	1:300	Dako GmbH, Hamburg, Germany
CD34	QBEnd-10	1:200	Antibodies-Online GmbH, Aachen, Germany
VEGF	VG76e	1:200	Antibodies-Online GmbH, Aachen, Germany
S100	Z0311	1:1000	Dako GmbH, Hamburg, Germany

### Termination of the experiment

The minipigs were anaesthetized as mentioned above. Final examination of bladder integrity, shape, and urinary reflux were performed via ultrasound and bladder filling with 0.9% sodium chloride solution.

The pigs were then euthanized by i.v. injection of 0.16 g/kg barbiturate (Narcoren^®^, Merial, Hallbergmoos, Germany). The urinary bladder and kidneys with ureters were removed and weighed. The urinary bladder was fixed for 24 h and kidneys with ureters were fixed for seven days in 4% phosphate buffered formaldehyde (Merck).

The whole bladder was processed for histological evaluation with emphasis on the implantation sites. Three sections of the kidney including cortex and medulla, the renal pelvis, as well as the proximal and distal ureters were examined for signs of pathological alterations. The implanted scaffolds were processed in the same manner as the cell-seeded scaffolds (described above). This involved sample dehydration, paraffin embedding and 3 µm sections being prepared. The histochemical staining of the bladder wall was performed using Elastica van Giesson’s staining (EVG) to provide an overview of the general morphology of the implantation site. PanCK was used to identify the urothelial layer, α-SMA was used for control of the detrusor muscle structure, cluster of differentiation 34 (CD34) and VEGF to identify blood vessels for vascularization and S100 as an indirect indicator of nerve fibre distribution (antibodies see Table 1). All staining methods were performed as mentioned above.

### Two-photon laser scanning microscopy (TPLSM)

In an attempt to evaluate the extent of implanted scaffold degradation, unstained sections were examined using a two-photon microscope (FV1000MPE, Olympus Corp., Tokyo, Japan) attached to a pulsed Ti–Sapphire laser (MaiTai DeepSee, SpectraPhysics) with a 25× water immersion objective (1.05NA, WD2.0). For excitation of the collagen remnants inside the tissue, the laser was tuned to the wavelength of 750 nm. The emission autofluorescent signals were collected with bandpass filters at 419–465, 495–540 and 590–650 nm, respectively. The intense autofluorescent structure of the scaffold was mainly detected in the range of 419–465 and 495–540 nm. Further image processing was performed using ImageJ (Version 1.49j10, National Institutes of Health, USA).

### Statistics

Data analysis of micturition volumes was performed using Origin (9.1 OG, 1991–2013 Origin Lab Corporation, Northampton, USA). All results are shown as mean ± SD. All examined data was tested for normal distribution by the Shapiro-Wilks test. Statistical differences were determined via One-way ANOVA with Tukey Post-Hoc test. P ≤ 0.05 was considered to be statistically significant.

## Results

### Seeding of scaffolds

**Fig. 4:**
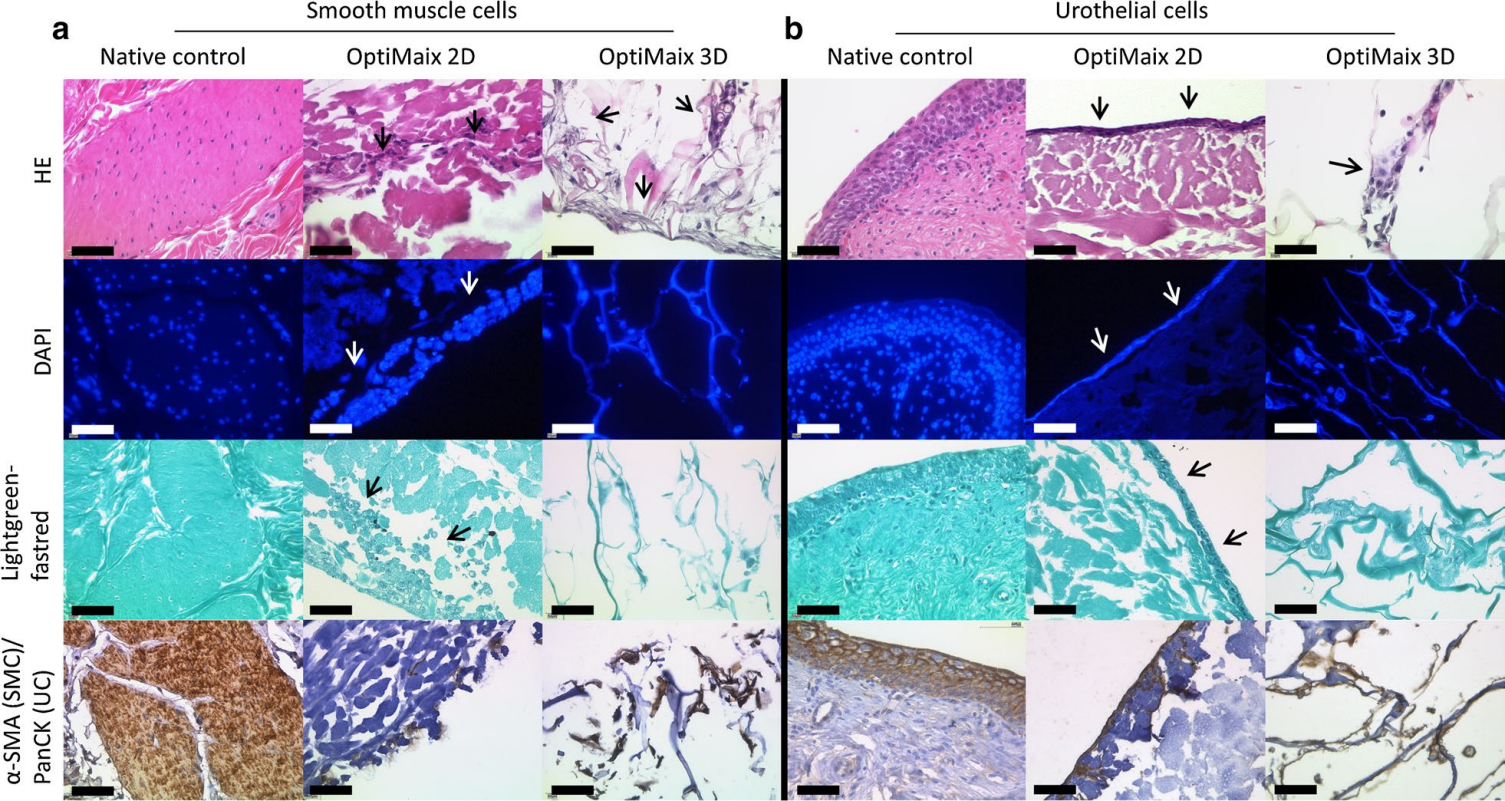
OptiMaix 2D and 3D seeded with (**a)** SMCs and (**b)** UCs before implantation. SMCs are building a multilayer on top of the fibres of the structured side of OptiMaix 2D (*arrows*) but are migrating inside the open pores of OptiMaix 3D. UCs are lining the dense side of OptiMaix 2D (*arrows*). Due to the open structure of OptiMaix 3D, UCs are also infiltrating the scaffold. *Scale bar* = 50 µm

Cell isolation resulted in differing cell cultivation counts based on intraindividual conditions of the animals. Nevertheless, all isolations were successful and a an average cell yield of 4.5 × 10^6^ UCs and 2.9 × 10^7^ SMCs could be seeded onto the scaffolds resulting in a distribution of 2.4 × 10^5^ (UCs) and 1.6 × 10^6^ (SMCs) per cm^2^. The SMCs were building a multilayer on top of the structured side of the OptiMaix 2D as the fibres of the scaffold were deflating when in contact with the cell culture medium (Fig. 4a). After 3 days, a closed layer of UCs was visible on the dense side of the OptiMaix 2D scaffold. After 2 weeks in culture, layers up to three cells thick could be observed in histological sections (Fig. 4b). The OptiMaix 3D scaffold allowed both UCs and SMCs to penetrate, adhere and grow inside the longitudinally orientated micro-pores of the 3D scaffold. Since these micro-pores had an average diameter of 100 µm, cells usually appeared to be associated with the walls of the porous framework rather than filling the pores.

### Surgery

The scaffold material of OptiMaix 2D had an appearance and texture similar to wet chamois and could, therefore, be easily sutured into the urinary bladder wall. In contrast, the OptiMaix 3D, was more delicate because of its highly porous structure. Preliminary attempts to suture the 3D scaffold in the same precut oval shape as the 2D failed due to rupturing of the scaffold at the suture points. This technical difficulty was overcome by using the whole 3 × 4 cm^2^ scaffolds that could be successfully sutured into the wall of the bladder. Instead of three small scaffolds, now only two original-sized 3D scaffolds were implanted.

**Fig. 5:**
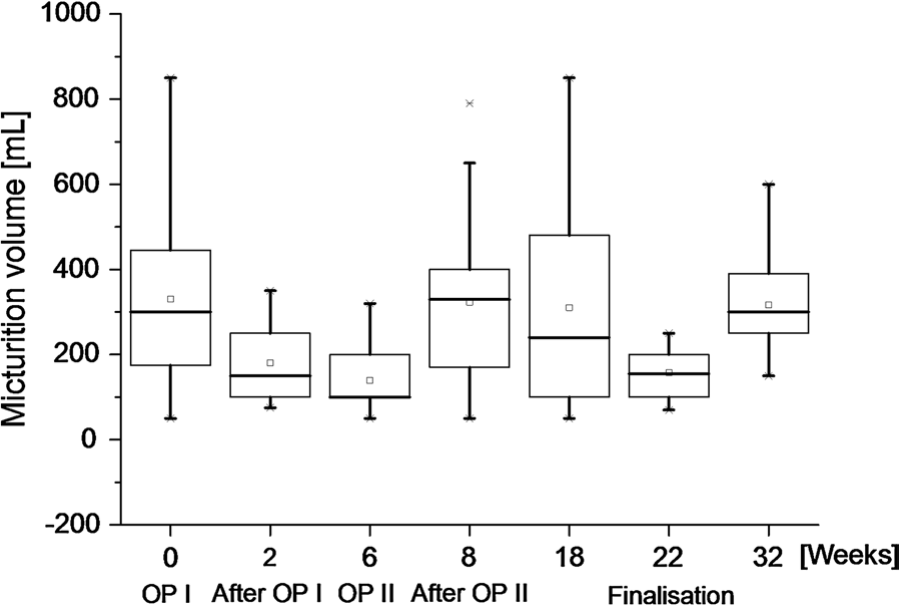
Micturition volume of six Göttingen minipigs at different stages of the experiment. The minipigs were monitored in a metabolic cage for 24 h with camera and flowmeter before each operation and after removal of the transurethral catheter. No significant differences in micturition volume could be detected between the different stages of the study

**Fig. 6:**
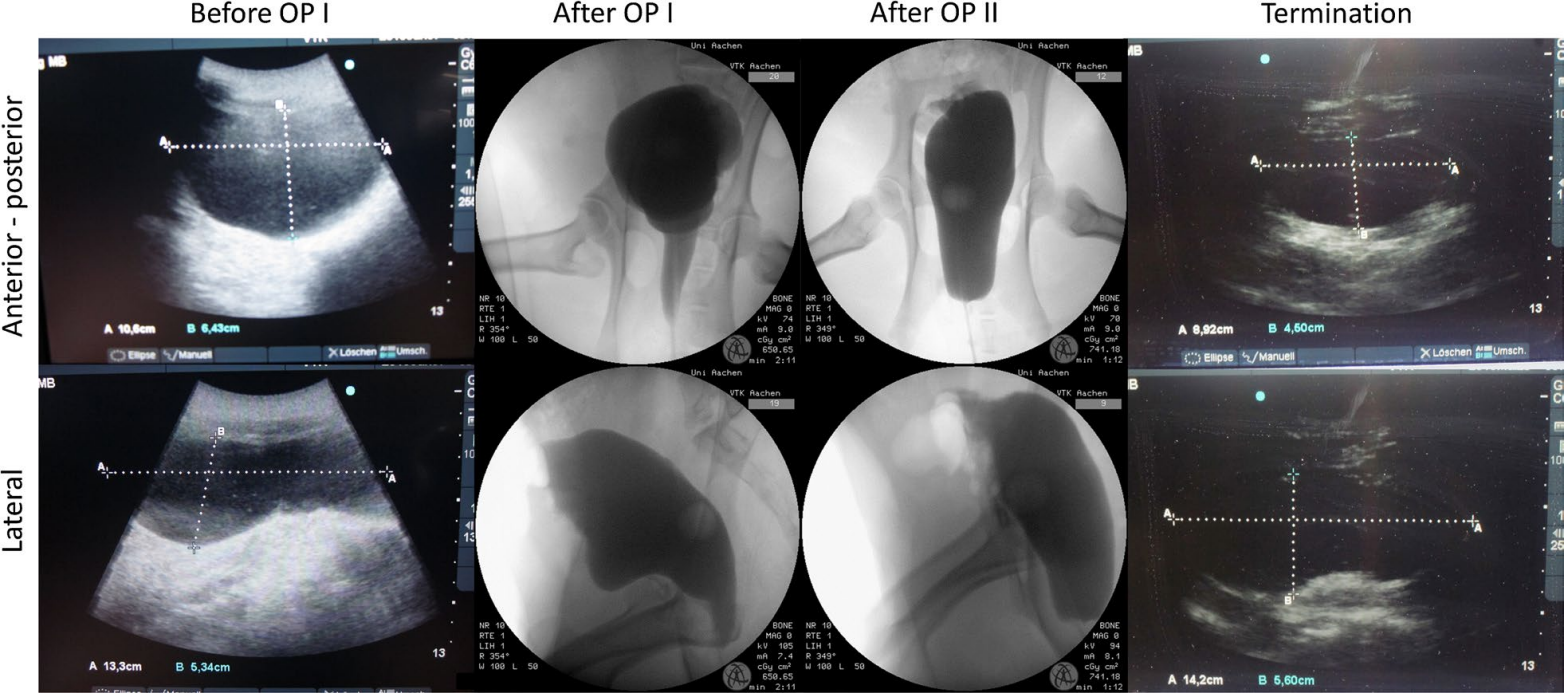
Ultrasound and cystography of Göttingen minipigs. Cystography was performed post-OP after removal of the transurethral catheter for evaluation of bladder shape and integrity. As the contrast agent lead to oedema, immediately before operations and the termination of the experiment only ultrasound was performed. Filling was performed with 300 ml sodium chloride (0.9%) and contrast agent respectively

All six animals recovered well after surgery and the analysis of blood parameters showed no signs of inflammation or infection. As a reaction to the blood loss that occurred during the operations, the number of thrombocytes had increased and haemoglobin and haematocrit values had decreased. These values returned to normal levels by the end of the experiment (Table 2). Although there was a tendency for reduced micturition volume at 6 weeks (the time point of OPII) there was no statistically significant reduction of volumes at any survival time studied (Fig. 5). There were no signs of incontinence or infections of the urinary tract. Cystography revealed that the implantation sites of the OptiMaix 3D scaffolds had a greater potential for leakages than the 2D scaffolds which occurred in two of the six pigs. The bladder shape was influenced by the multiple implants leading to slightly pear-shaped urinary bladders. However, by the end of the experiment, the bladder demonstrated a normal shape (Fig. 6). There was no urinary reflux to the kidneys. Before each operation and at the termination of the experiment, only ultrasound was performed, since the contrast agent lead to oedema in the bladder urothelium.

**Table 2:** Blood and renal parameters of Göttingen minipigs

	OP I	After OP I	OP II	After OP II	Termination	Norm
Kidney values (serum)	0 weeks	2 weeks	6 weeks	8 weeks	18/22/32 weeks	
Total protein (g/dl)	6.4 ± 0.7	5.2 ± 0.5	6.7 ± 0.5	6.1 ± 0.3	6.5 ± 0.7	4.5–8.5
Urea (mmol/l)	2.4. ± 1.1	1.8 ± 0.1	2.2 ± 0.9	3.7 ± 3.7	2.2 ± 0.9	3.3–8.3
Creatinine (µmol/l)	107.5 ± 13,8	78.4 ± 16.7	119.3 ± 20.8	127.8 ± 73.6	107.7 ± 19.5	40–133
Uric acid (µmol/l)	14.0 ± 0.7	13.8 ± 0.8	13.5 ± 0.8	14.5 ± 1.8	14.0 ± 0	–
Sodium (mmol/l)	143.8 ± 1.7	141.2 ± 1.3	141.7 ± 8.3	144.0 ± 1.7	142.5 ± 2.7	140–160
Potassium (mmol/l)	3.7 ± 0.3	3.7 ± 0.4	3.6 ± 0.7	3.9 ± 0.5	3.7 ± 0.3	4.0–5.0
Chloride (mmol/l)	101.5 ± 2.1	99.0 ± 1.4	100.0 ± 0.9	99.0 ± 2.4	101.5 ± 0.6	102–106
*Blood count*						
Leucocytes (×10^3^/µl)	7.2 ± 1.6	7.5 ± 2.1	7.6 ± 4.7	11.9 ± 6.7	7.5 ± 4.0	7.0–22.0
Erythrocytes (×10^6^/µl)	5.8 ± 0.7	5.0 ± 0.9 ↓	7.1 ± 2.3	4.5 ± 1.1 ↓	5.5 ± 0.9	5.8–8.0
Thrombocytes (×10^3^/µl)	507.7 ± 93.1	699.0 ± 179.9 ↑	371.7 ± 171.3	819.3 ± 236.8 ↑	554.0 ± 142.0	175–587
Haemoglobin (g/dl)	11.6 ± 1.1	10.5 ± 1.7	14.2 ± 4.1	9.2 ± 1.9 ↓	11.1 ± 0.9	10.0–16.0
Haematocrit (%)	32.2 ± 2.3	29.1 ± 4.2 ↓	39.0 ± 10.9	25.8 ± 5.3 ↓	30.9 ± 2.5	33.0–45.0
MCV (fl)	56.0 ± 6.1	58.3 ± 4.7	56.2 ± 6.1	57.8 ± 6.1	56.8 ± 5.6	50.0–68.0
MCH (pg)	20.2 ± 2.2	20.9 ± 1.8	20.5 ± 2.3	20.5 ± 2.3	20.4 ± 2.2	17.0–21.0
MCHC (g/dl)	36.2 ± 0.9	35.9 ± 1.0	36.4 ± 0.6	35.5 ± 0.7	36.0 ± 0.6	30.0–35.0
*Differential blood count*						
Unsegmented granulocytes (%)	5.0 ± 1.9	5.8 ± 1.9	5.8 ± 3.1	5.3 ± 2.2	5.2 ± 3.6	0–7
Segmented granulocytes (%)	31.5 ± 16.4	33.4 ± 11.4	33.7 ± 14.3	36.2 ± 12.5	26.5 ± 18.5	10–39
Lymphocytes (%)	54,5 ± 17,6	51.2 ± 10.1	54.2 ± 15.1	43.8 ± 22.7	60.5 ± 24.7	49–85
Monocytes (%)	8.0 ± 3.5	7.6 ± 2.3	5.5 ± 1.4	7.0 ± 2.6	6.7 ± 2.7	0–5
Eosinophils (%)	0.7 ± 1.0	1.8 ± 0.8	0.7 ± 0.8	0.7 ± 1.0	1.0 ± 0.9	0–6
Basophils (%)	0.3 ± 0.5	0.2 ± 0.4	0.2 ± 0.4	0.3 ± 0.5	0.2 ± 0.4	0–2

All results are shown as mean ± SD

No significant differences could be examined

### Histological evaluation

The serosa mainly was a homogenous closed layer, solely, the usage of different coloured non-degradable marking sutures allowed a macroscopic differentiation of OptiMaix 2D and 3D scffolds. However, the marking sutures favoured adhesion of the bladder serosa to the omentum and surrounding organs and had to be carefully dissected during the removal of the urinary tract at the end of the experiment. Bladder weight was in the range of 89.5 ± 16.2 g, the left kidney had an average weight of 105.5 ± 22.2 g and the right kidney of 108.5 ± 26.8 g. After 32 weeks, the implantation sites were still visible on the interior surface. No macroscopic anomalies of the urinary tract were visible. Kidneys and ureters were without any pathological alteration, no kidney reflux occurred and the bladder was without calcifications or stone formation.

**Fig. 7:**
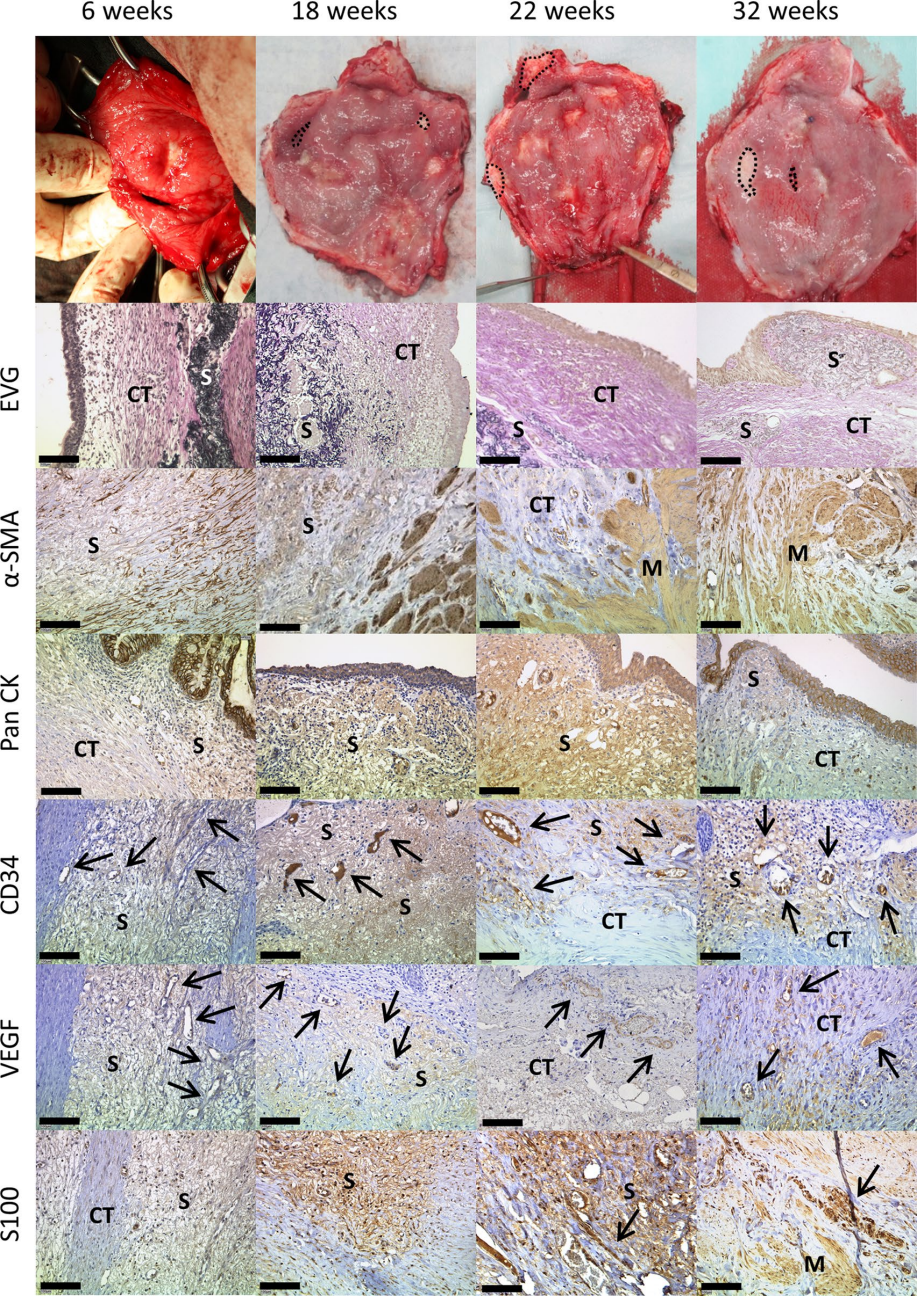
Histological development of implanted unseeded OptiMaix 2D scaffolds. Six weeks after implantation, the scaffold structure is still dense, but degradation has already started and is proceeding until 32 weeks. Connective tissue is forming on top of the scaffold (EVG) and is lined by newly built urothelium (PanCK). Blood vessels are growing into the artificial collagen (CD34, VEGF) and nerve fibres (S100), which go together with the ingrowth of adjacent muscle tissue (α-SMA). *S* scaffold, *M* muscle, *CT* connective tissue. *Scale bar* = 50 µm

**Fig. 8:**
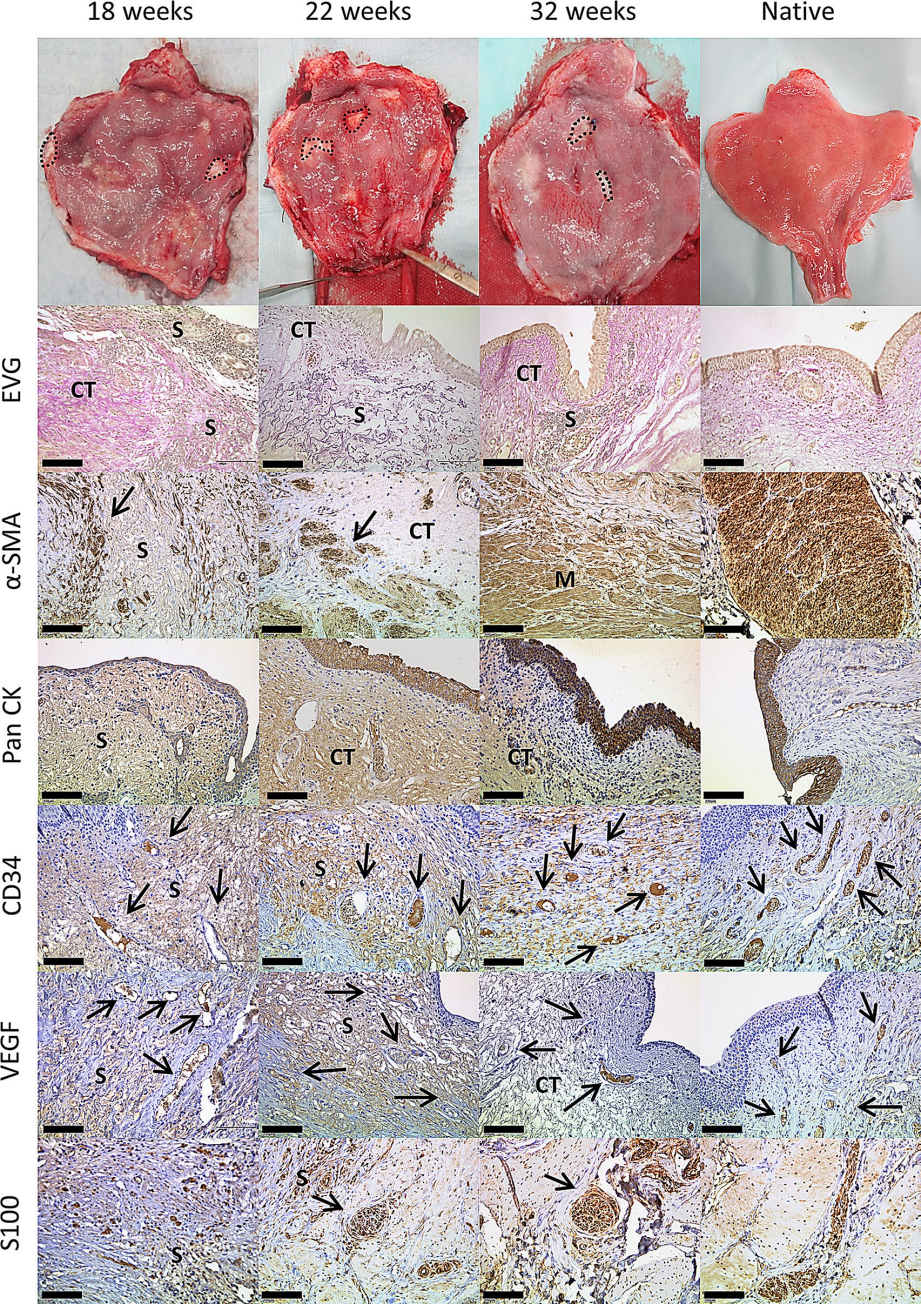
Histological development of implanted seeded OptiMaix 2D scaffolds. Eighteen weeks after implantation, like in the unseeded scaffold, still dense regions of scaffold tissue can be found (EVG), but are already degrading and are broken up by the surrounding tissue and infiltrated by blood vessels (CD34, VEGF). Building of an urothelial layer and connective tissue can also be noticed (EVG, PanCK). Muscle tissue together with nerve fibres is growing in from the adjacent tissue (S100, α-SMA). Overall, the development of the implantation sites until 32 weeks is comparable to the unseeded OptiMaix 2D scaffolds. *S* scaffold, *M* muscle, *CT* connective tissue. *Scale bar* = 50 µm

At week six, the scaffold structure seemed nearly intact but the degradation process had already started. Remnants of the OptiMaix 2D were still visible after 32 weeks. Figures 7 and 8 show this process, as the foreign collagen appears dark violet in the EVG staining. However at this time, a closed urothelial layer with underlying connective tissue was clearly visible on the inner surface of the OptiMaix 2D scaffold. The seeding of urothelial cells on the collagen scaffold here did not seem to have any influence on this process. The detrusor muscle grew from the sides of the implant towards its centre. Here, the dense structure decelerated the ingrowth of the adjacent tissue into the scaffold. Furthermore, pre-seeding with SMCs did not improve the ingrowth process. Nevertheless, until 32 weeks, muscle tissue was growing. The staining with CD34 and VEGF showed enhanced vascularization with blood vessels growing through the scaffold material at six and 18 weeks. This vascularization seemed to become less with 22 weeks and was at a normal level compared to the native control when the tissue had reorganized itself at 32 weeks. The S100 staining showed a continuous ingrowth of nerve fibres into the new tissue.

**Fig. 9:**
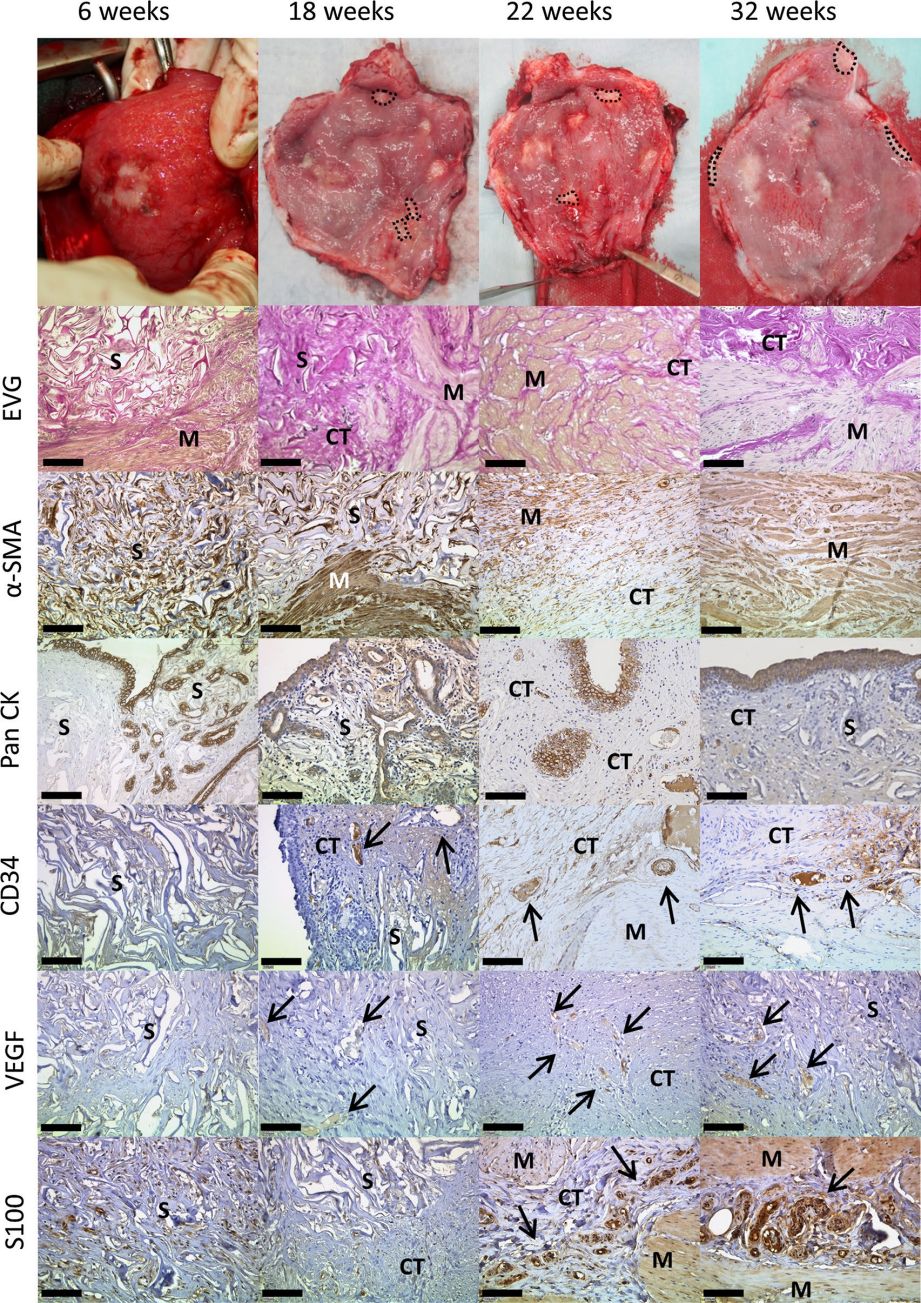
Histological development of implanted unseeded OptiMaix 3D scaffolds. Six weeks after implantation, the scaffold seems to be still intact. Connective tissue has started to grow inside the open pores (EVG) and also the ingrowth of muscle tissue is occurring (α-SMA). The urothelium has closed the luminal side of the scaffold but is growing scattered and building vesicles inside the pores (PanCK). These processes are advancing until week 32 with building of a more structured bladder wall and a smoother urothelium. Blood vessels (CD34, VEGF) and nerve fibres (S100) too, are evolving during this time span. *S* scaffold, *M* muscle, *CT* connective tissue. *Scale bar* = 50 µm

**Fig. 10:**
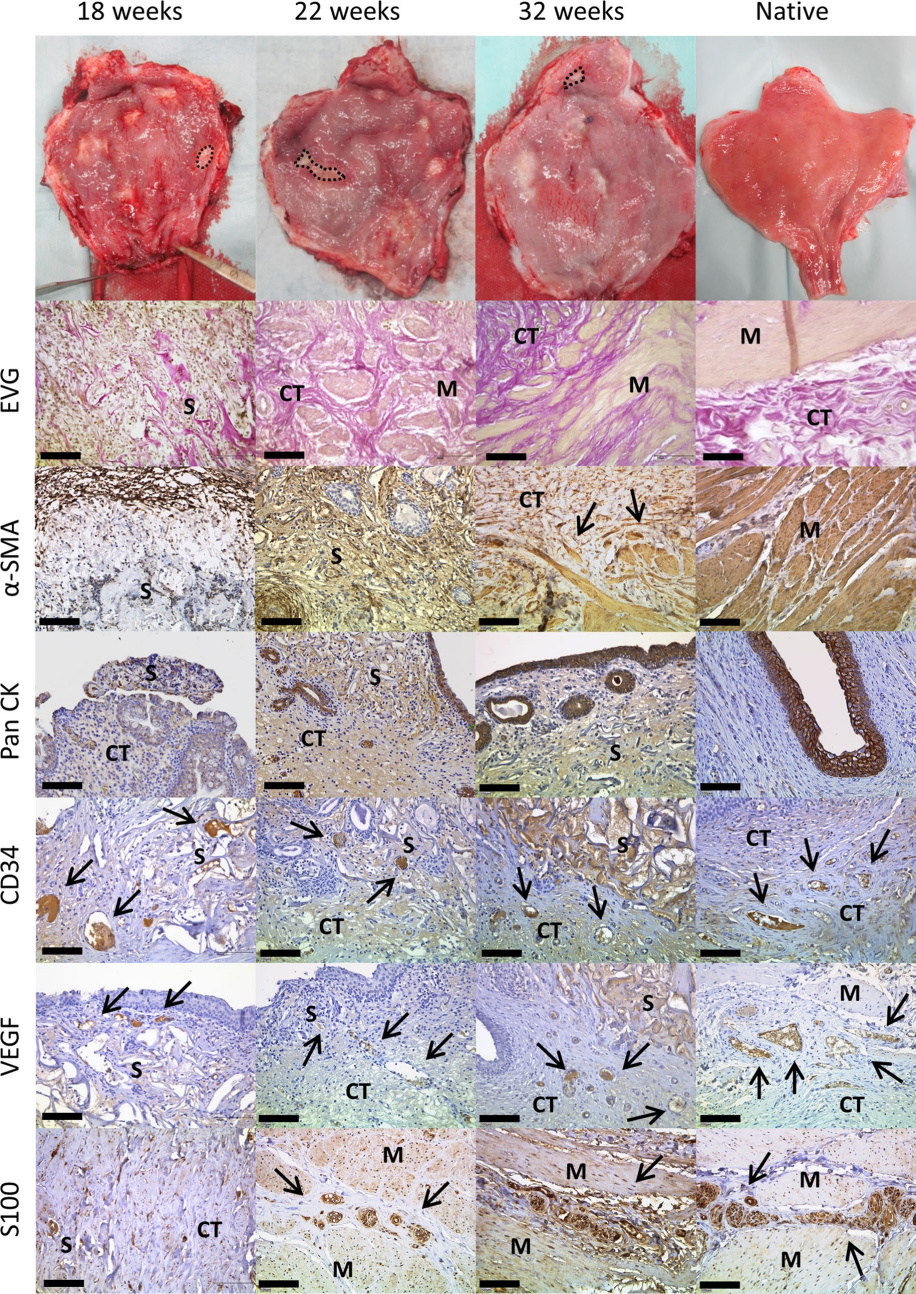
Histological development of implanted seeded OptiMaix 3D scaffolds. Similar to the unseeded OptiMaix 3D, the degradation has started after 6 weeks, but large pieces of the scaffold can still be found. At 18 weeks, parts of the scaffold structure are encapsulated by fibrotic tissue (α-SMA), which might be due to the protein component of the cell culture medium. This phenomenon cannot be found at later time points. As in the unseeded scaffolds, the urothelium is closed at 18 weeks and gradually building a smooth layer like in the native tissue. Overall, no differences can be found between the building of a new urothelial lining (PanCK), the degradation of the scaffolds (EVG), blood vessels (CD34, VEGF) or nerve fibre ingrowth (S100) compared to the unseeded scaffold. *S* scaffold, *M* muscle, *CT* connective tissue. *Scale bar* = 50 µm

OptiMaix 3D remnants could also be found after 32 weeks (Figs. 9, 10). Compared to the dense structure of the 2D scaffold, after 6 weeks, adjacent connective tissue had grown inside the wide pores of the sponge-like structure. Seeding with cells in this case also did not accelerate this process, but quite the contrary; the seeded 3D scaffolds seemed to be encapsulated by the surrounding tissue in both pigs at 18 weeks. This phenomenon could not be found in the four pigs housed for 22 and 32 weeks. Here, remnants of the scaffold could favourably be found beneath the urothelium and less in the muscle regeneration zone. As anticipated, the pore structure of the OptiMaix 3D was a disadvantage concerning the urothelium. The urothelial cells, like in vitro, lined the inside of these pores and were not able to build a closed layer on top of the scaffolds. Instead, scattered urothelial clusters had formed inside the scaffold by 6 weeks. This phenomenon was increasing until 18 weeks and abated after 22 weeks. At 32 weeks, the urothelium on top of the scaffold had reorganized in association with ongoing scaffold-degradation, and could clearly be seen as a single, continuous band of cells. Vascularization and nerve-fibre ingrowth were comparable to the OptiMaix 2D scaffold.

**Fig. 11:**
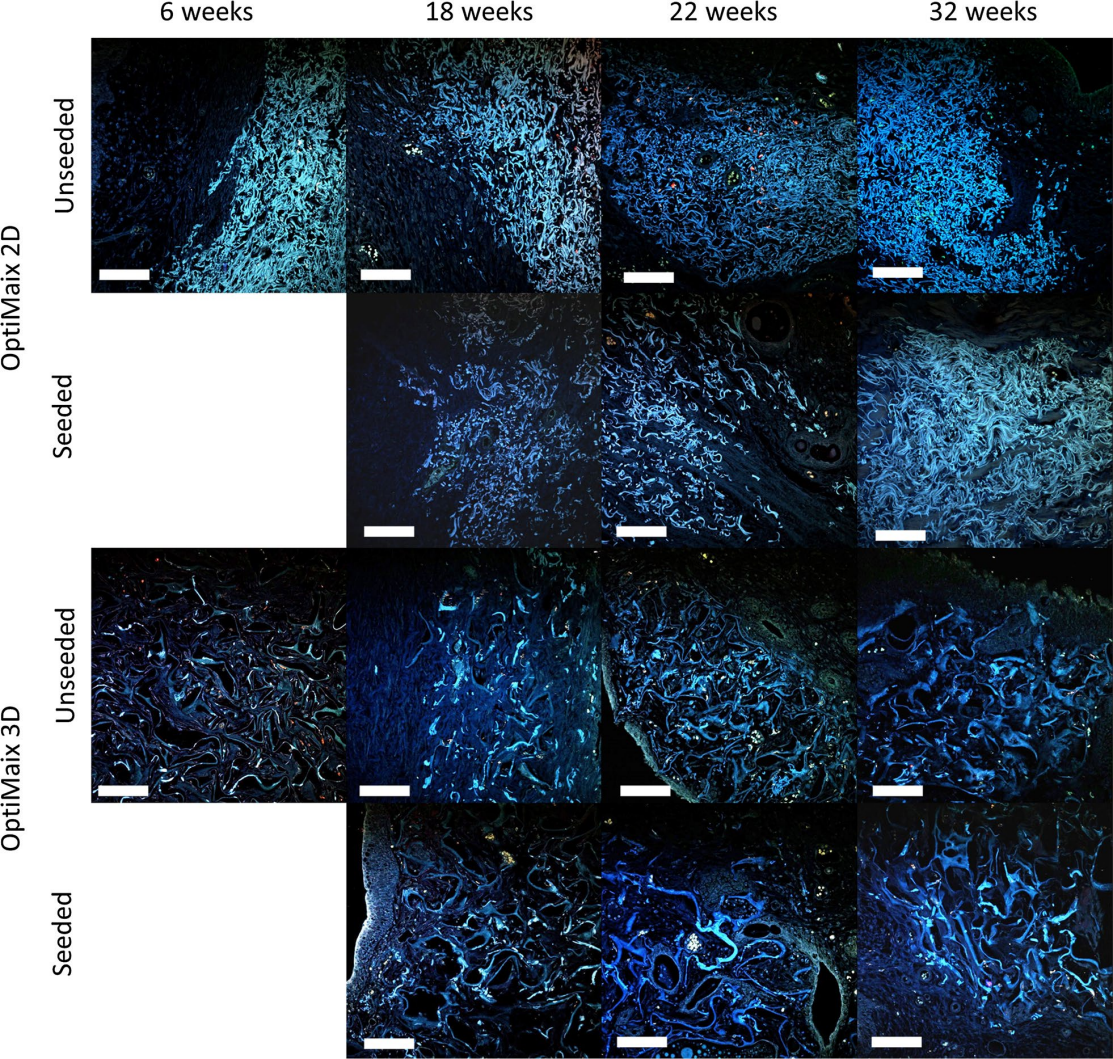
TPLSM—autofluorescence of unseeded (*above*) and seeded (*below*) OptiMaix scaffolds. After 6 weeks, 2D still appears compact but is degrading and infiltrated by the adjacent bladder tissue. The 3D scaffold enhances the ingrowth of connective and muscle tissue due to its sponge-like structure. After 32 weeks, large pieces of both OptiMaix scaffold types are still detectable in the peripheral regions on the luminal side of the implantation sites. *Scale bar* = 100 µm

TPLSM confirmed the general histological and immunohistochemical observations concerning the degradation of the OptiMaix scaffolds. Remnants could be mainly found in the occlusion zone in the periphery of the implantation sites (Fig. 11). The ingrowth of adjacent tissue into the OptiMaix 2D was clearly less prominent than in the OptiMaix 3D. Yet again, no difference between seeded and unseeded scaffolds could be detected.

## Discussion

The aim of this study was the comparison of two differently manufactured collagen scaffolds OptiMaix as material for bladder wall repair. In this context, the building of a (multi-)layer of cells on both scaffolds with a subsequent good cell growth in vitro speaks for a high biocompatibility. The dense side of the OptiMaix 2D reached a desirable result for the UCs, whereas, the SMCs were only growing on the surface of the scaffold instead of growing in. The OptiMaix 3D in contrast, allowed migration of cells into the scaffold structure, which is not desirable for the UCs. Another issue arising with the OptiMaix 3D was the difficult sewing process during the operations. Regarding this aspect, the OptiMaix 2D was relatively easier to handle and additionally one week after operation, the implantation sites of the 2D scaffolds were closed. Indicated by cystographies, the implantation sites of the 3D scaffolds needed longer recovery; hence, the pigs were in need of a transurethral catheter for an extended period. These differences in permeability coincide with the findings of Montzka et al. [32] who performed experiments with these scaffolds in an Ussing chamber.

Six pigs with big inter-individual micturition differences were used in our study. Although there were differences between the micturition volumes before and after the operations, these were not significant and at the end point of the experiment all animals had regained normal bladder function. Furthermore, we had no indications for reflux, incontinence or graft failure which are common problems in urinary tissue engineering studies [19, 38].

The flexibility of the OptiMaix 3D allowed a temporary dilatation of the bladder wall before the recovery process had started and the ingrowing tissue stabilized the implantation sites. As this is the first time that these scaffolds are tested in vivo for urinary bladder augmentation, this is a critical outcome.

We did not find any significant indications that seeding with autologous bladder cells had improved ingrowth into the bladder wall. Here, the small size of the scaffolds together with the sealing with serosa might have promoted the ingrowth of urothelium and detrusor muscle from the adjacent tissue resulting in a greater impact on the outcome than the seeding with cells. Additionally, the seeded 3D scaffolds, contrary to the unseeded, were encapsulated by fibrotic tissue at 6 weeks. This might be due to the fact that the cell-scaffold hybrids were incubated in cell culture medium containing FCS. The seeded 2D scaffolds did not show any signs of encapsulation which might be due to the smaller overall surface that also reduces the possibility for foreign proteins to adhere [39]. Also, the degradation of both scaffolds was not accelerated when seeded with autologous cells.

The vascularization process seemed elevated for the OptiMaix 2D scaffolds compared to 3D. Already after 6 weeks, first blood vessels had formed within the compact material. No inflammation response regarding the scaffolds occurred, which permits the conclusion of a good biocompatibility in vivo. This is further supported by the absence of stone- or biofilm-formation. Overall, the bladder function after the implantation was physiologic and even as 25% of the bladders are augmented. Not to mention, the bladder is withstanding the pressure and the scaffold implants whatsoever, are participating in the bladder function.

By TPLSM, it was clearly visible that after six weeks the OptiMaix 3D was much more fragmented compared to the 2D. The degradation process, for both scaffolds, was not completed at 26 weeks. Especially in the fringe of the implantation site, remnants of the scaffolds could be detected, notably beneath the urothelium.

We are very well aware of the limitations of our study. A future approach should entail a partial cystectomy and therefore, the fabrication of a larger prototype is definitely necessary. Also, as we were already proposing in our in vitro study [37], a combination of both scaffolds could be advantageous and provide a more convenient support for both cell types in vitro and in vivo and lead to a more stable implantation site.

In this study, we only used healthy pigs and therefore cannot make a statement about the ingrowth of the material into morbid tissue. Similarly, the behaviour of urothelial cells isolated from a diseased bladder is impaired, which has to be taken into consideration [6, 40]. The next logical step would be the use of animals with a diseased bladder, e.g. idiopathic detrusor over activity or bladder exstrophy [19, 41, 42].

## Conclusions

With this study, we were able to show that the OptiMaix scaffolds are very promising for bladder wall augmentation. On the one hand, OptiMaix 2D seems more favourable compared to its 3D counterpart regarding handling, easier implantation, a quicker tightness of the bladder, and better reaction of the surrounding tissue. On the other hand, the ingrowth of cells and tissue into the 3D is facilitated and a combination of both scaffold types could be promising. Nevertheless, the usage for bigger defects might include the necessity for seeding with cells, which has to be evaluated in future studies.

## Abbreviations

α-SMA: α-smooth muscle actin
BAMG: bladder acellular matrix graft
CD34: cluster of differentiation 34
DAPI: 4′,6-Diamidin-2-phenylindol
EVG: Elastica van Giesson
FCS: fetal calf serum
HE: haematoxylin–eosin
IKVAV: isoleucine–lysine–valine–alanine–valine
MEM: modified Eagle’s medium
OPI: operation I
OPII: operation II
PanCK: pancytokeratin
PCL: polycaprolactone
PGA: polyglycolic acid
PLA: polylactic acid
RG: nuclear fast red–fast green
RGD: arginine–glycine–aspartic acid
SIS: small intestinal submucosa
SMC: smooth muscle cell
SPF: specific pathogen free
TPLSM: two photon laser scanning microscopy
UC: urothelial cells
VEGF: vascular endothelial growth factor
